# International Lower Limb Collaborative Paediatric subpopulation analysis (INTELLECT-P) study: multicentre, international, retrospective audit of paediatric open fractures

**DOI:** 10.1093/bjsopen/zrae082

**Published:** 2024-07-27

**Authors:** Anna Y Allan, Juan E Berner, James K Chan, Matthew D Gardiner, Jagdeep Nanchahal, Abhilash Jain, INTELLECT Collaborative, A Navia, R Tejos, A Ortega-Briones, H A Rakhorst, G Nolan, H Samarendra, A Mohan, K Cooper, J Skillman, A Kennedy, A Qureshi, K Wallis, L Harry, A Hagiga, S Ibrahim, M Albendary, K A Shah, C B Chuo, C Katsura, J R Rodríguez Astudillo, A López Ortega, J P Henríquez Rissios, M Nova Nova, J Hughes, C Wearn, D Peberdy, B Ho, K Gohil, A Abood, N Rabey, M Nizamoglu, G Biosse-Duplan, K To, S R Sabapathy, M Mohan, H Venkatramani, S Rajasekaran, H Hsu, A R Ambriz Plascencia, L E Escalona Ramírez, C A Zepeda Torres, E Santamaria, S Vallejo Toro, C West, W Bhat, C McArdle, S Louette, S Hassan, P W van Egmond, W J J Bekkers, D Capitani, L Troisi, T Talamonti, P Capitani, V Cerbone, G Materazzi, L Ballini, J Tomas-Hernandez, J A Porcel-Vazquez, Y Garcia-Sanchez, J V Andrés-Peiró, J Teixidor-Serra, J Selga-Marsà, H Dafydd, S Ali, R Slade, S Tarassoli, B Olías López, J Boluda Mengod, D González Martín, A Bashir, A Dearden, V Itte, F Smith, C W Lee, V A A Paulus, P Romijn, T N Tromp, T de Jong, A Rodríguez, E L Jonsson, S Holm, O Wolff, A Abugarja, H Elbahari, H K S Hamid, M Awadelkarim, J Erdocia Pascual, L Bahillo O’Mahoney, M A Quiroga Bilbao, M Felipe Peña, W Eardley, A Egglestone, S Taher, N Wei, J Martínez Ros, G Valero Cifuentes, A Ondoño Navarro, A Escudero Martínez, A Ortega Columbrans, P Zamora, J Masiá, A Ibarra, M Fernández, V Giblin, A Kilshaw, B Wood, M Wyman, I E Tinhofer, E Seidl, C J Tzou, S Quadlbauer, J Reichetseder, H Bürger, T Hausner, S van Miltenburg, I Beijk, W Verra, R de Groot, A Crick, C Mitchell, T Curran, R Kuo, S Eltoum Elamin, P Caba Doussoux, D Alonso Tejero, J Gómez Alcaraz, J M Pardo García, K Kooi, R Poelstra, J P Hong, M Jang, D W Hong, J G Kwon, M Francés Monasterio, J Fernández-Palacios Martínez, A Suarez Cabañas, M Marrero Martínez-Carlón, W ten Cate, J E D Jacobs, J Palma, A Cuadra, H Demandes, S Canahuate, D Moreno, S Norton, J Thompson, G Lafford, D Noriego Muñoz, A Teixido de la Cruz, M Vázquez Gómez, W Ayad, A Elbatawy, M Ouf, M Cherubino, L Garutti, G Molina Olivella, A Endemaño Lucio, R Moral-Nestares, F Requena, M A Giraldez, R Moreno Domínguez, B Martínez Sañudo, A Robinson, C Digney

**Affiliations:** Plastic Surgery Department, Guy’s and St Thomas’ Hospital NHS Foundation Trust, London, UK; Kellogg College, University of Oxford, Oxford, UK; Plastic Surgery Department, Royal Victoria Infirmary, Newcastle upon Tyne, UK; Nuffield Department of Orthopaedics, Rheumatology and Musculoskeletal Sciences (NDORMS), University of Oxford, Oxford, UK; Plastic Surgery Department, Stoke Mandeville Hospital, Aylesbury, UK; Nuffield Department of Orthopaedics, Rheumatology and Musculoskeletal Sciences (NDORMS), University of Oxford, Oxford, UK; Plastic Surgery Department, Wexham Park Hospital, Slough, UK; The Kennedy Institute of Rheumatology, University of Oxford, Oxford, UK; Nuffield Department of Orthopaedics, Rheumatology and Musculoskeletal Sciences (NDORMS), University of Oxford, Oxford, UK; Plastic Surgery Department, Imperial College Healthcare NHS Trust, London, UK

## Introduction

Trauma is a leading cause of mortality and morbidity in the paediatric population^1^. Available epidemiological data on paediatric lower limb trauma are scarce^2,3^. The International Lower Limb Collaborative (INTELLECT) study aimed to describe the treatment and outcomes of these injuries from a global perspective. The authors present an analysis of the paediatric population included in the INTELLECT study, representing the largest global study on paediatric open lower limb trauma.

## Methods

The INTELLECT study^2^ was a STROBE-compliant, international, multicentre, retrospective audit supported by the Reconstructive Surgery Trials Network. Investigators in participating centres (*Supplementary Methods*) retrieved demographic and clinical data for patients admitted with open lower limb fractures between 1 January 2017 and 31 December 2018. Exclusion criteria were patients whose definitive treatment was performed at a different unit to the participating centre, open forefoot fractures, and isolated patella fractures. Primary outcomes were soft tissue infection, deep infection, non-union, and amputation. Secondary outcomes were time to discharge and instances of deep-vein thrombosis. Each centre was given a list of outcome definitions to ensure consistency. Each collaborating centre was asked to validate 2% of their cases. Records were exported to an encrypted Microsoft^®^ Excel (Microsoft Corporation, Redmond, WA, USA) spreadsheet. Missing data were quantified for each variable.

For this paediatric group, the age of inclusion was 0–17 years at the time of injury. Children aged 5 years and below were analysed and children aged below 12 years and 12 years or older were analysed to capture the prepubertal subgroup and assess differences with teenagers. The paediatric data were included in the previously published INTELLECT study^2^. Statistical analyses were performed using Microsoft^®^ Excel. ORs with 95% confidence intervals were calculated and *P* < 0.050 was considered statistically significant.

## Results

A total of 43 centres in 13 countries contributed 163 patients, a median of 6 per unit (*Table 1*). The median age at presentation was 13 (range 2–17) years, with 63 patients (39%) aged under 12 years and 100 patients (61%) aged 12 years and above, and 70% of patients were male. The most common mechanism of injury was a road traffic accident (71%), followed by a high-energy fall (28%) and a low-energy fall (1%). Most injuries were open tibial/fibula fractures (80%), followed by open foot fractures (10%) and open femoral fractures (9%). In total, 6% of patients had co-morbidities (40% of patients who had co-morbidities had asthma). The median follow-up was 11 (range 0–43, interquartile range 5–18) months.

**Table 1:** Descriptive baseline information uploaded to the INTELLECT-P database

	Total (*n* = 163)	Femoral fractures (*n* = 15)	Tibial/fibular fractures (*n* = 131)	Foot fractures (*n* = 17)
Age (years), median	13	16	13	13
**Sex**				
Male	114 (70)	12 (80)	93 (71)	9 (53)
Female	49 (30)	3 (20)	38 (29)	8 (47)
**Mechanism of injury (*n* = 163)**				
Road traffic accident	116 (71)	14 (93)	89 (67)	13 (76)
High-energy fall (>2 m)	45 (28)	1 (7)	41 (31)	3 (18)
Low-energy fall (from standing or seating)	2 (2)	0 (0)	1 (2)	1 (6)
**Fracture classification (Gustilo–Anderson) (*n* = 153)**				
I	32 (21)	3 (20)	25 (20)	4 (27)
II	41 (27)	8 (53)	30 (24)	3 (20)
IIIA	26 (17)	4 (27)	20 (16)	2 (13)
IIIB	43 (28)	0 (0)	37 (30)	6 (40)
IIIC	11 (7)	0 (0)	11 (9)	0 (0)
**Patients per country**				
UK	66	3	61	2
Mexico	29	1	25	3
Spain	25	5	14	6
Netherlands	10	1	9	0
Chile	6	3	3	0
Sudan	6	2	4	0
Egypt	6	0	4	2
South Korea	4	0	1	3
Italy	4	0	3	1
Taiwan	2	0	2	0
India	2	0	2	0
Austria	2	0	2	0
Sweden	1	0	1	0
**Co-morbidities**				
None	153 (94)	14 (93)	123 (94)	16 (94)
Asthma	4 (2)	1 (7)	2 (1)	1 (6)
Not specified	6 (4)	0 (0)	6 (5)	0 (0)
**Smoker**				
Yes	15 (9)	3 (20)	10 (8)	2 (12)
No	127 (78)	7 (47)	106 (81)	14 (82)
Unknown	21 (13)	5 (33)	15 (11)	1 (6)

Values are *n* (%) unless otherwise indicated.

Approximately one-third of open tibial/fibular fractures that required soft tissue reconstruction were classified as Gustilo–Anderson IIIB and IIIC after debridement. In patients with Gustilo–Anderson IIIB and IIIC injuries, there was a higher risk of wound infection (OR 4.16 (95% c.i. 1.35 to 12.88); *P* = 0.013) and deep infection (OR 27.29 (95% c.i. 1.52 to 489.90); *P* = 0.025). There was no statistically significant association between sex, time to antibiotics, or time to primary wound excision and soft tissue infection, deep infection, non-union, or amputation. A delay in achieving soft tissue closure beyond 72 h after injury (41 patients) was associated with a greater likelihood of soft tissue infection (OR 14.67 (95% c.i. 1.25 to 171.73); *P* = 0.032) and deep infection (OR 1.97 (95% c.i. 0.09 to 41.45); *P* = 0.66). Only 14% of patients were treated by a combination of orthopaedic and plastic surgeons, from the time of debridement onwards.

A total of three patients required amputation, all of whom were involved in road traffic accidents and were 12 years of age or older. Of the amputations, two were performed within 24 h of injury. The other patient had an early amputation, between 24 h and 3 months post-injury. Younger patients aged 5 years and below (13 patients) were more likely to develop soft tissue infection (OR 5.19 (95% c.i. 0.81 to 33.15); *P* = 0.082) and deep infection (OR 16.89 (95% c.i. 2.36 to 120.67); *P* = 0.005). All 9 patients with non-union were aged 12 years or older (median of 16 (range 13–17) years). Treatment and outcome data are presented in *Table 2*.

**Table 2:** Treatment and outcome data uploaded to the INTELLECT-P database

	Total (*n* = 163)	Femoral fractures (*n* = 15)	Tibial/fibular fractures (*n* = 131)	Foot fractures (*n* = 17)
Time to antibiotics (h), median	2	1	2	2
Time to primary wound excision (h), median	11	5	12	11
Time to primary wound excision (h), mean	22	12	20	44
**Specialties involved in primary wound excision (*n* = 163)**				
Orthopaedic surgeons	114 (70)	13 (87)	88 (67)	13 (76)
Plastic surgeons	19 (12)	1 (7)	17 (13)	1 (6)
Orthopaedic and plastic surgeons	23 (14)	0 (0)	20 (15)	3 (18)
Trauma surgeons	7 (4)	1 (7)	6 (5)	0 (0)
**Seniority of surgeon leading primary wound excision (*n* = 161**)				
Consultant level	115 (71)	12 (80)	90 (70)	13 (76)
Non-consultant level	46 (29)	3 (20)	39 (30)	4 (24)
Time to definitive skeletal fixation (days), median	1.0	6.5	1.0	1.0
**Primary mode of definitive skeletal fixation (*n* = 163)**				
Casting	16 (10)	0 (0)	12 (9)	4 (24)
Ex-fix frame	29 (18)	0 (0)	29 (22)	0 (0)
Ex-fix rods and pins	6 (4)	0 (0)	6 (5)	0 (0)
IMN	42 (26)	11 (73)	31 (24)	0 (0)
Kirschner wires	19 (12)	0 (0)	11(8)	8 (47)
Plate and screws	43 (26)	3 (20)	35 (27)	5 (29)
Other	8 (5)	1 (7)	7 (5)	0 (0)
Soft tissue reconstruction required	61 (38)	1 (7)	52 (41)	8 (47)
**Modality of soft tissue closure (*n* = 60)**				
Dressings	1 (2)	0 (0)	1 (2)	0 (0)
TNP	4 (7)	0 (0)	4 (8)	0 (0)
SSG only	13 (22)	1 (100)	10 (20)	2 (25)
Local flap	3 (5)	0 (0)	3 (6)	0 (0)
Regional flap	8 (13)	0 (0)	8 (16)	0 (0)
Free flap	31 (52)	0 (0)	25 (49)	6 (75)
Time to soft tissue closure (days), median	6	5	6	12.5
**Flap failure/survival rate (*n* = 43)**				
Total flap failure	2 (5)	0 (0)	2 (5)	0 (0)
Partial flap failure	1 (2)	0 (0)	1 (3)	0 (0)
Total flap survival	40 (93)	0 (0)	34 (92)	6 (100)
Deep-vein thrombosis (*n* = 163)	0 (0)	0 (0)	0 (0)	0 (0)
Wound infection (*n* = 162)	20 (12)	1 (7)	17 (13)	2 (12)
Time to wound infection (days), median	17	17	25	8
Deep infection (*n* = 163)	8 (5)	0 (0)	8 (6)	0 (0)
Time to deep infection (days), median	13	0	13	0
Non-union (*n* = 160)	9 (6)	1 (7)	8 (6)	0 (0)
**Amputation (*n* = 159)**				
Immediate	2 (1)	0 (0)	2 (2)	0 (0)
Early	1 (1)	0 (0)	0 (0)	1 (6)
Late	0 (0)	0 (0)	0 (0)	0 (0)
Time to discharge (days), median	9	13	9	9
Follow-up (months), median	11	12	11	6

Values are *n* (%) unless otherwise indicated. IMN, intramedullary nail; TNP, topical negative pressure; SSG, split-thickness skin graft.

## Discussion

To the authors’ knowledge, this is the largest report on outcomes of paediatric open fractures. Infective complications in the cohort were consistent with previous reports^4–6^, whilst subgroup analysis demonstrated higher rates of wound infection and deep infection in children aged 5 years and below^7,8^. An association was found between a delay in achieving soft tissue closure beyond 72 h and both soft tissue infection and deep infection.

Approximately one-third of paediatric cases requiring soft tissue reconstruction were graded as Gustilo–Anderson IIIB or IIIC. Whilst the Gustilo–Anderson classification has high inter-observer variability^9^, this may not be the only factor. This discrepancy was also seen in the INTELLECT cohort, although it was half the proportion seen in the paediatric cohort^2^. The greater discrepancy in patients aged 5 years and below may be due to a tendency to limit the extent of tissue excised at primary debridement. The rates of wound infection and deep infection in this paediatric series were higher than for adults in the INTELLECT study^2^. This may reflect different risk factors for and susceptibility to surgical-site infections in the paediatric population, particularly in those aged 5 years and below, which is well described in the paediatric surgical literature, but not for open fractures^10,11^. A possible explanation is that surgeons consider that children have a greater healing potential and, therefore, fail to perform adequate primary soft tissue excision^5^, underestimating the Gustilo–Anderson grading.

The rate of non-union was greater in patients aged 12 years and older, with 78% of these aged 16 years and above. None of these patients required amputation within the data collection window. This non-union rate was consistent with previously reported findings^4,5^ and may be influenced by increased osteogenic capacity documented in patients below the age of 10 years^12,13^.

Only 14% of patients were managed using a combined ortho-plastic approach, despite guidelines^14,15^ and evidence for reduced infection rates^16,17^. This may be due to paediatric major trauma centres not always being co-located with adult major trauma centres, a lack of guideline adherence, and global variation in practice.

This study has the limitations of a retrospective design; the applicability to middle- and low-income settings is limited, as the majority of cases were from the UK. A prospective study would allow more reliable data collection with longer follow-up that could include mental health and quality-of-life patient-reported outcome measures, such as the Children’s Revised Impact of Events Scale (CRIES)^18^ and Paediatric Quality of Life Inventory (PedsQL)^19^, to ensure more holistic outcome capture for children and young people with lower limb trauma.

Paediatric open fractures are a surgical priority and remain the leading cause of global disability-adjusted life years in adolescents^20^. This study provides global data on expected outcomes for paediatric lower limb trauma. The data highlight that the severity of soft tissue injuries must not be underestimated in the paediatric population.

Soft tissue injuries in children with open fractures are challenging to manage, especially as the majority are related to high-energy trauma from road traffic accidents. The authors’ findings support their previous recommendation^5^, the updated Standards for the Management of Open Fractures^15^ and the more recent National Institute for Health and Care Excellence (NICE) guidance^14^ that the soft tissue envelope in paediatric trauma should be managed with a combined ortho-plastic approach, meticulous primary wound excision to remove all non-viable tissue, and, when needed, reconstruction with a vascularized flap within 72 h of the injury.

## Collaborators

**INTELLECT Collaborative**

A. Navia, R. Tejos, A. Ortega-Briones, H. A. Rakhorst (INTELLECT Steering Committee); G. Nolan, H. Samarendra, A. Mohan, K. Cooper (Imperial College Healthcare NHS Trust, London, UK); J. Skillman, A. Kennedy, A. Qureshi, K. Wallis (University Hospitals Coventry and Warwickshire NHS Trust, Coventry, UK); L. Harry, A. Hagiga, S. Ibrahim, M. Albendary, K. A. Shah (Brighton and Sussex University Hospitals NHS Trust, Brighton, UK); C. B. Chuo, C. Katsura (Hull University Teaching Hospital NHS Trust, Hull, UK); J. R. Rodríguez Astudillo, A. López Ortega, J. P. Henríquez Rissios, M. Nova Nova (Hospital Sótero del Río, Santiago, Chile); J. Hughes, C. Wearn, D. Peberdy, B. Ho, K. Gohil (University Hospitals Plymouth NHS Trust, Plymouth, UK); A. Abood, N. Rabey, M. Nizamoglu, G. Biosse-Duplan, K. To (Cambridge University Hospitals NHS Foundation Trust, Cambridge, UK); S. R. Sabapathy, M. Mohan, H. Venkatramani, S. Rajasekaran (Ganga Hospital, Coimbatore, India); H. Hsu (Dalin Tzu Chi Hospital—Buddhist Tzu Chi Medical Foundation, Dalin, Taiwan); A. R. Ambriz Plascencia, L. E. Escalona Ramírez, C. A. Zepeda Torres (Hospital Civil de Guadalajara, Guadalajara, Mexico); E. Santamaria, S. Vallejo Toro (Hospital General ‘Dr. Manuel Gea González’, Mexico City, Mexico); C. West, W. Bhat, C. McArdle, S. Louette, S. Hassan (Leeds Teaching Hospitals NHS Foundation Trust, Leeds, UK); P. W. van Egmond, W. J. J. Bekkers (Elisabeth-TweeSteden Ziekenhuis, Tilburg, Netherlands); D. Capitani, L. Troisi, T. Talamonti, P. Capitani, V. Cerbone, G. Materazzi, L. Ballini (ASST Grande Ospedale Metropolitano Niguarda, Milan, Italy); J. Tomas-Hernandez, J. A. Porcel-Vazquez, Y. Garcia-Sanchez, J. V. Andrés-Peiró, J. Teixidor-Serra, J. Selga-Marsà (Vall d’Hebron Barcelona Hospital Campus, Barcelona, Spain); H. Dafydd, S. Ali, R. Slade, S. Tarassoli (Morriston Hospital—Swansea Bay University Health Board, Swansea, UK); B. Olías López, J. Boluda Mengod, D. González Martín (Hospital Universitario de Canarias, Santa Cruz de Tenerife, Spain); A. Bashir, A. Dearden, V. Itte, F. Smith, C. W. Lee (The Newcastle upon Tyne Hospitals NHS Foundation Trust, Newcastle, UK); V. A. A. Paulus, P. Romijn, T. N. Tromp, T. de Jong (Radboud UMC, Nijmegen, Netherlands); A. Rodríguez, E. L. Jonsson, S. Holm, O. Wolff (Uppsala University Hospital, Uppsala, Sweden); A. Abugarja, H. Elbahari, H. K. S. Hamid, M. Awadelkarim (Ribat University Hospital and Ibrahim Malik Teaching Hospital, Khartoum, Sudan); J. Erdocia Pascual, L. Bahillo O’Mahoney, M. A. Quiroga Bilbao, M. Felipe Peña (Complejo Hospitalario Universitario Insular Materno Infantil, Las Palmas de Gran Canaria, Spain); W. Eardley, A. Egglestone, S. Taher, N. Wei (South Tees Hospitals NHS Foundation Trust, Middlesbrough, UK); J. Martínez Ros, G. Valero Cifuentes, A. Ondoño Navarro, A. Escudero Martínez, A. Ortega Columbrans (Hospital Clínico Universitario ‘Virgen de la Arrixaca’, Murcia, Spain); P. Zamora, J. Masiá, A. Ibarra, M. Fernández (Hospital de la Santa Creu i Sant Pau, Barcelona, Spain); V. Giblin, A. Kilshaw, B. Wood, M. Wyman (Sheffield Teaching Hospitals NHS Foundation Trust, Sheffield, UK), I. E. Tinhofer, E. Seidl, C. J. Tzou (Hospital of Divine Savior, Vienna, Austria); S. Quadlbauer, J. Reichetseder, H. Bürger, T. Hausner (AUVA Traumazentrum, Vienna, Austria); S. van Miltenburg, I. Beijk, W. Verra, R. de Groot (Medisch Spectrum Twente, Enschede, Netherlands); A. Crick, C. Mitchell, T. Curran, R. Kuo, S. Eltoum Elamin (Salisbury NHS Foundation Trust, Salisbury, UK); P. Caba Doussoux, D. Alonso Tejero, J. Gómez Alcaraz, J. M. Pardo García (Hospital 12 de Octubre, Madrid, Spain); K. Kooi, R. Poelstra (Medisch Centrum Leeuwarden, Leeuwarden, Netherlands); J. P. Hong, M. Jang, D. W. Hong, J. G. Kwon (Asan Medical Center, Seoul, South Korea); M. Francés Monasterio, J. Fernández-Palacios Martínez, A. Suarez Cabañas, M. Marrero Martínez-Carlón (Hospital Universitario de Gran Canaria ‘Doctor Negrín’, Las Palmas de Gran Canaria, Spain); W. ten Cate, J. E. D. Jacobs (Ziekenhuisgroep Twente, Hengelo, Netherlands); J. Palma, A. Cuadra, H. Demandes, S. Canahuate, D. Moreno (Hospital Clínico Pontificia Universidad Católica de Chile, Santiago, Chile); S. Norton, J. Thompson, G. Lafford (Norfolk and Norwich University Hospitals NHS Foundation Trust, Norwich, UK); D. Noriego Muñoz, A. Teixido de la Cruz, M. Vázquez Gómez (Hospital Universitari Josep Trueta, Girona, Spain); W. Ayad, A. Elbatawy, M. Ouf (Al Azhar Hospital, Cairo, Egypt); M. Cherubino, L. Garutti (Universita degli studi dell’Insubria, Varese, Italy); G. Molina Olivella, A. Endemaño Lucio (Hospital Consorci Sanitari Moises Broggi, Barcelona, Spain); R. Moral-Nestares, F. Requena (Hospital Universitario San Cecilio, Granada, Spain); M. A. Giraldez, R. Moreno Domínguez, B. Martínez Sañudo (Hospital Universitario Vírgen del Rocío, Sevilla, Spain); A. Robinson, C. Digney (Ulster Hospital—South Eastern Health and Social Care Trust, Dundonald, UK).

## Supplementary Material

zrae082_Supplementary_Data

## Data Availability

The data that support the findings of this study are not openly available due to reasons of sensitivity and are available from the corresponding author upon reasonable request.
