# Supplementary material for: International Lower Limb Collaborative Paediatric subpopulation analysis (INTELLECT-P) study: multicentre, international, retrospective audit of paediatric open fractures
Source: BJS Open. 2024 Jul 27;8(4):zrae082. doi: 10.1093/bjsopen/zrae082 (PMC11282435; doi:10.1093/bjsopen/zrae082)
Supplement: zrae082_Supplementary_Data [file zrae082_supplementary_data.docx]

**International Lower Limb Collaborative Paediatric subpopulation analysis (INTELLECT-P) study: multi-centre, international, retrospective audit of paediatric open fractures**

**Authors: Anna Yarlini Allan^1^*, Juan Enrique Berner^2,3^*, James K Chan^4,5^, Matthew D Gardiner^4,6^, Jagdeep Nanchahal^7^, Abhilash Jain^4,8^**

**Affiliations:**

1. **Guy’s and St Thomas’ Hospital NHS Foundation Trust. London, UK**
2. **Kellogg College, University of Oxford. Oxford, UK**
3. **Plastic Surgery Department, Royal Victoria Infirmary. Newcastle, UK**
4. **Nuffield Department of Orthopaedic, Rheumatology and Musculoskeletal Science (NDORMS), University of Oxford. Oxford, UK**
5. **Plastic Surgery Department, Stoke Mandeville Hospital. Aylesbury, United Kingdom**
6. **Plastic Surgery Department, Wexham Park Hospital. Slough, United Kingdom**
7. **The Kennedy Institute of Rheumatology, University of Oxford. Oxford, UK**
8. **Imperial College Healthcare NHS Trust. London, UK**

***Contributed equally to this work**

**Supplementary Materials - Index**

| **Supplementary Methods** |  |
| --- | --- |
| Participating centers | *pages 2-3* |
| Primary and secondary outcome definitions | *page 4* |
|  |  |

**Supplementary Methods**

**Participating centres**

| City, Country | Participating centre |
| --- | --- |
| Brighton, UK | Brighton and Sussex University Hospitals NHS Trust |
| Cambridge, UK | Cambridge University Hospitals NHS Foundation Trust |
| Coventry, UK | University Hospitals Coventry and Warwickshire NHS Trust |
| Plymouth, UK | University Hospitals Plymouth NHS Trust |
| Hull, UK | Hull University Teaching Hospital NHS Trust |
| London, UK | Imperial College Healthcare NHS Trust |
| Leeds, UK | Leeds Teaching Hospitals NHS Foundation Trust |
| Middlesbrough, UK | South Tees Hospitals NHS Foundation Trust |
| Newcastle, UK | The Newcastle upon Tyne Hospitals NHS Foundation Trust |
| Norwich, UK | Norfolk and Norwich University Hospitals NHS Foundation Trust |
| Salisbury, UK | Salisbury NHS Foundation Trust |
| Sheffield, UK | Sheffield Teaching Hospitals NHS Foundation Trust |
| Swansea, UK | Morriston Hospital - Swansea Bay University Health Board |
| Dundonald, UK | Ulster Hospital - South Eastern Health and Social Care Trust |
| Cairo, Egypt | Al Azhar Hospital |
| Seoul, South Korea | Asan Medical Center |
| Las Palmas de Gran Canaria, Spain | Complejo Hospitalario Universitario Insular Materno Infantil |
| Barcelona, Spain | Hospital Consorci Sanitari Moises Broggi |
| Sevilla, Spain | Hospital Universitario Vírgen del Rocío |
| Madrid, Spain | Hospital 12 de Octubre |
| Barcelona, Spain | Hospital de la Santa Creu i Sant Pau |
| Murcia, Spain | Hospital Clínico Universitario ‘Virgen de la Arrixaca’ |
| Santa Cruz de Tenerife, Spain | Hospital Universitario de Canarias |
| Las Palmas de Gran Canaria, Spain | Hospital Universitario de Gran Canaria ‘Doctor Negrín’ |
| Granada, Spain | Hospital Universitario San Cecilio |
| Girona, Spain | Hospital Universitari Josep Trueta |
| Barcelona, Spain | Vall d'Hebron Barcelona Hospital Campus |
| Tilburg, Netherlands | Elisabeth-TweeSteden Ziekenhuis |
| Leeuwarden, Netherlands | Medisch Centrum Leeuwarden |
| Enschede, Netherlands | Medisch Spectrum Twente |
| Nijmegen, Netherlands | Radboud UMC |
| Hengelo, Netherlands | Ziekenhuisgroep Twente |
| Dalin, Taiwan | Dalin Tzu Chi Hospital - Buddhist Tzu Chi Medical Foundation |
| Coimbatore, India | Ganga Hospital |
| Guadalajara, Mexico | Hospital Civil de Guadalajara |
| Mexico City, Mexico | Hospital General ‘Dr. Manuel Gea González’ |
| Santiago, Chile | Hospital Sótero del Río |
| Santiago, Chile | Hospital Clínico Pontificia Universidad Católica de Chile |
| Khartoum, Sudan | Ribat University Hospital and Ibrahim Malik Teaching Hospital |
| Milan, Italy | ASST Grande Ospedale Metropolitano Niguarda |
| Varese, Italy | Universita degli studi dell'Insubria |
| Uppsala, Sweden | Uppsala University Hospital |
| Vienna, Austria | AUVA Traumazentrum |

**Primary and secondary outcome definitions**

| *Wound infection* | Defined as redness and swelling, with or without discharge, that was treated with antibiotics. If surgery was performed, no evidence of infection was found deep to muscle fascia. |
| --- | --- |
| *Deep tissue infection* | Defined as infections below muscle fascia, including bone, that require surgical exploration for lavage, removal of metalwork and/or further bone debridement. |
| *Osteomyelitis* | Proven bone infection based on clinical, radiographic or microbiological findings. |
| *Non-union* | Lack of union requiring unplanned surgical intervention after definitive wound closure or incomplete radiographic healing at 1 year. |
